# Progression and Transformation of Clonally Heterogeneous B-cell Lymphoma

**DOI:** 10.1371/journal.pone.0130590

**Published:** 2015-06-12

**Authors:** Robert F. Weiss, Mitchell R. Smith, Merlin G. Miller, John F. Cronin

**Affiliations:** 1 Computational Biology, Back Bay Biosciences LLC, Boston, Massachusetts, United States of America; 2 Hematology and Oncology, The Cleveland Clinic, Cleveland, Ohio, United States of America; 3 Computer Services, Physical Sciences Inc., Andover, Massachusetts, United States of America; IRCCS National Cancer Institute, ITALY

## Abstract

Indolent B- cell non-Hodgkin lymphoma can transform into aggressive lymphoma. We extend our prior mathematical model to analyze and predict transformation. To provide additional confidence in our model, we compare it with SCID mouse data for combination therapy of Diffuse Large B-cell Lymphoma, an aggressive form of the disease. We develop a two cell model that includes indolent and aggressive clones but no immune response and use it to predict transformation. An approximate model for the time to transformation is derived that provides insight regarding scaling effects. We then add an immune response and therapeutic measures, and illustrate the complex interactions among the various processes, with a focus on transformation. The implications for initial diagnosis and treatment of non-Hodgkin lymphoma are discussed.

## Introduction

Indolent B- cell non-Hodgkin lymphoma (iNHL) can transform into aggressive lymphoma. In Follicular Lymphoma (FL), the most common iNHL, this occurs in up to 30% of cases over the course of the disease, and is a major cause of death from FL. The analogous process in chronic lymphocytic leukemia (CLL), another indolent B cell disease that is the most common leukemia in Western populations, is called Richter’s transformation. Transformation has generally been described as a stochastic event in which the indolent cells acquire new mutations, a “second-hit”, conferring enhanced growth and/or treatment resistance properties. While this stochastic model accounts for the often abrupt clinical change and the fairly constant annual incidence, it does not explain why significantly reducing the number of cells at risk with effective treatment does not reduce the risk of transformation, nor the more recent finding that FL transformation almost never occurs after 15 years.

Most tumors are clonally heterogeneous, composed of more and less aggressive clones. In CLL, deep sequencing has demonstrated that a low level of presumably aggressive sub-clones containing mutations in p53 can be present at the time of diagnosis yet not become clinically apparent until later in the disease course [1]. Even extremely small initial populations of highly proliferative cells can eventually overtake the less aggressive cells, transforming CLL from indolent to clinically aggressive behavior [2,3].

Here, we extend our prior mathematical model [4], previously used to analyze SCID mouse data for combination therapy of FL, to evaluate transformation. We demonstrate that an acute change in clinical behavior at various times in the disease course can result from a process of clonal evolution favoring varying amounts of aggressive clones present at the time of diagnosis, as opposed to a “second-hit”. Our updated model includes indolent and aggressive B-cell clones as well as an active immune system.

To provide additional confidence in our model, we compare it with SCID mouse data for combination therapy of Diffuse Large B-cell Lymphoma (DLBCL), the most common aggressive sub-group of NHL and the usual result of transformation of FL and CLL. This comparison further validates the model in the aggressive subtypes of NHL and illustrates some of the essential differences between indolent and aggressive NHL. This then permits us to model the interaction between indolent and aggressive clones in a heterogeneous population [5]. Even a model that ignores immune effects yields information on disease transformation.

Finally, we develop a three cell model that includes indolent and aggressive clones, as well as an immune response, in the presence of therapeutic measures, and illustrate the complex interactions among these processes, with a focus on transformation. The implications for initial diagnosis and treatment of NHL are discussed.

## Two-cell Model

Our model [4,5] describes the population history of malignant B-cells in the presence of healthy T-cells, and includes the effects of environmental competition and both direct and indirect B-cell kill by therapeutic agents and cytotoxic T-cells. When the model is applied to mice the defining equations are:

$${\text{dN*}}_{\text{B}}{\text{ / dt* = N*}}_{\text{B}}{\text{\{1 - K*}}_{\text{B}}{\text{[1 + N*}}_{\text{B}}{\text{ + N*}}_{\text{T}}{\text{ + f(d*)(K" + K'''N*}}_{\text{T}}\text{)]\}}$$ (1)

dN* T/ dt* = BN*T{1 - K*T[1 + N*B + N*T]} (2)

where:

t* = non-dimensional time = t K_Bb_

K_Bb_ = B-cell birth rate

N_B_(0) = initial malignant B—cell population in mice

N*_B_ = B-cell number / N_B_ (0)

N*_T_ = T-cell number / N_B_ (0)

K*_B_ = B-cell death rate / B-cell birth rate = K_Bd_ / K_Bb_

K' = K*_B_ therapeutically modified

K*_T_ = T-cell death rate / T-cell birth rate = K_Td_ / K_Tb_

K'' = drug induced B-cell kill rate/B-cell death rate = K_k_ / K_Bd_

f (d*) = dependence of kill rate (K_k_) on relative drug dosage (d* = d/d_m_)

d_m_ = minimum effective dosage

K''' = T-cell induced B-cell kill rate / B-cell death rate = K_Tk_ / K_Bd_

B = T-cell birth rate / B-cell birth rate = K_Tb_ / K_Bb_

The ratio of malignant B-cell death rates to birth rates, K*_B_, is a number that is small compared to unity. When this ratio is modified by the introduction of anti-Bcl-2 drugs; e.g., ABT-263 or ABT-199, we designate it as K'. The direct kill rate of anti-CD-20 monoclonal antibodies compared to the malignant cell death rate is designated as K'', and the ratio of T-cell assisted kill rate to malignant cell death rate is defined as K'''. We determine the values for these parameters from control and mono-therapy experiments, and then predict the result of combination therapy with our model.

## Three-cell Model

B-cell populations can be heterogeneous with multiple cell types. This can be modeled by adding an equation similar to Eq (1) for each cell type. When applied to humans, N_L_, the total number of lymphocytes in humans, is substituted for N_B_(0) in the normalization of all cell populations. In the case of two clones (indolent and aggressive) we obtain a three cell model:

$${\text{dN*}}_{\text{i}}{\text{/dt* = N*}}_{\text{i}}{\text{ \{1 - K*}}_{\text{i}}{\text{ [1 + N*}}_{\text{i}}{}_{\;}{\text{+ N*}}_{\text{a}}{\text{ + N*}}_{\text{T}}{\text{ + f(d*)(K''+K''' N*}}_{\text{T}}\text{ )] \}}$$ (3)

$${\text{dN*}}_{\text{a}}{\text{/dt* = A N*}}_{\text{a}}{\text{ \{1- K*}}_{\text{a}}{\text{ [1 + N*}}_{\text{i}}{\text{ + N*}}_{\text{a}}{\text{ + N*}}_{\text{T}}{\text{ + f(d*)(K''+K'''N*}}_{\text{T}}\text{ )] \}}$$ (4)

$${\text{dN*}}_{\text{T}}{\text{/dt* = B N*}}_{\text{T}}{\text{ \{1-K*}}_{\text{T}}{\text{ [1 + N*}}_{\text{a}}{\text{ + N*}}_{\text{i}}{\text{ +N*}}_{\text{T}}\text{ ]\}}$$ (5)

The parameters in these equations are the same as those defined in the two-cell model defined above. The subscripts “i” and “a” indicate indolent and aggressive cell types, respectively. To derive these equations, we only had to add the aggressive cell number N*_a_ to the sum N*_B_ + N*_T_ and identify N*_B_ as N*_i_. One new parameter, A, is required to complete the model: A is the ratio of the aggressive to indolent B-cell birth rates.

## Comparison with SCID Mouse Data

**Fig 1:**
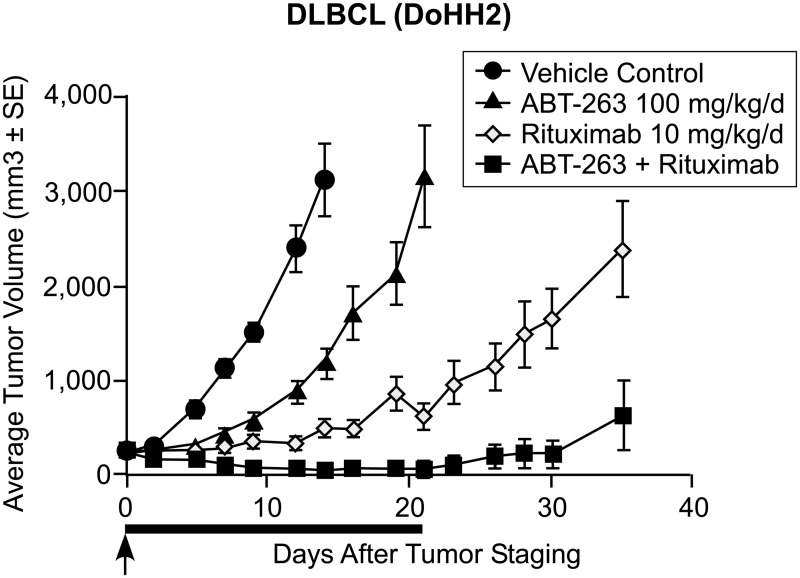
SCID mice treated with ABT-263 and rituximab independently and in combination (Reprinted from [6] under a CC BY license, with permission from Cancer Research, original copyright 2008).

Previously [4], we compared our model to data for anti-Bcl-2 therapy combined with rituximab, an anti-CD-20 drug. Here, we utilize SCID mouse data (N*_T_ = 0) obtained with ABT-263 [6], an anti-Bcl-2 drug predecessor to ABT-199 [7], reproduced with permission in Fig 1. These data correspond to Diffuse Large B-Cell Lymphoma, an aggressive NHL, whereas our prior model/data comparisons were for FL, an indolent lymphoma.

**Fig 2:**
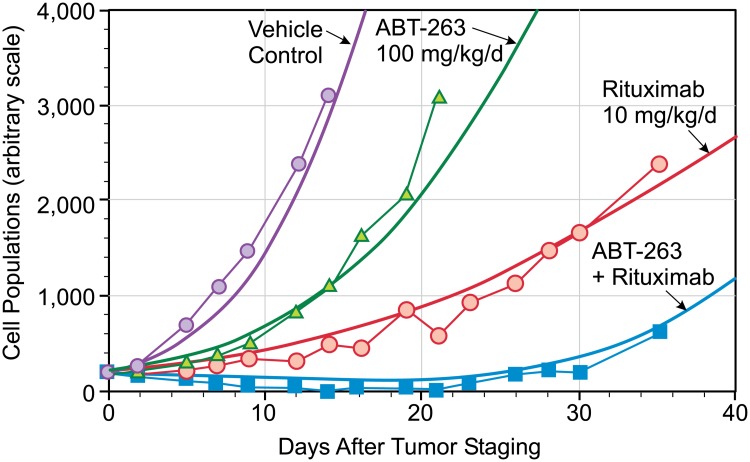
Model calculations compared to data, with combination therapy prediction using parameters derived from monotherapy data.

We derive the relationship between t* and dimensional time (days) by fitting our model (Eq 1) to the Vehicle control data. In aggressive disease, the malignant cell birth rate is higher than in indolent disease, and its characteristic time (t* = 1) is 4 days. With this parameter fixed, we then determine K' by fitting the ABT-263 data, and derive K'' from the rituximab results. In the absence of T-cells, we cannot determine the value of K''' for any dosage f (d*). Finally, we predict the time history of the combination data (ABT-263 and rituximab) with these parameters fixed. Each of these model calculations is presented in Fig 2.

We find that K' is 0.02, which is consistent with a birth rate that is higher than that for indolent lymphomas (e.g., FL) and a death rate that is also typical of all B-cell lymphomas. We also find that the product K'' f (d*) = 24, derived from the rituximab mono-therapy data, is an order of magnitude larger than previously derived for an indolent lymphoma [2]. With data for only a single dosage we cannot separate these two factors.

As seen in Fig 2, the predicted population history with combination therapy is in excellent agreement with the combination therapy data if the K'' f(d*) product is increased to 30 for the first 20 days and then reduced to zero in the last 10 days. This indicates that the combination of ABT-263 and rituximab is more effective than either therapy alone, again consistent with our previous analysis, but its effectiveness is of limited duration.

## Disease Transformation Model

In the absence of an effective immune system (N*_T_ = 0), our model of a heterogeneous population reduces to a two equation model for indolent and aggressive populations N*_i_ and N*_a_, respectively.

$${\text{dN*}}_{\text{i}}{\text{ / dt* = N*}}_{\text{i}}{\text{ \{1 - K*}}_{\text{i}}{\text{ [1+ N*}}_{\text{i}}{\text{+ N*}}_{\text{a}}\text{ + f(d*) K" ] \}}$$ (6a)

$${\text{dN*}}_{\text{a}}{\text{ / dt* = AN*}}_{\text{a}}{\text{ \{1 - K*}}_{\text{a}}{\text{ [1 + N*}}_{\text{i}}{\text{ + N*}}_{\text{a}}\text{ + f(d*) K" ] \}}$$ (6b)

Note that with the exception of the different values of K* or K' and the parameter A, the ratio of aggressive to indolent B-cell birth rates, these equations are identical. However, they do not require that an immune response or therapeutic action be equally effective with either clone, as the parameters K'' will typically have different values for the two clones.

Ignoring an immune system response will not only model future experiments with SCID mice, but may also be appropriate for humans in clinical cases where the immune system is incapable of killing indolent B-cells, much less aggressive cells. It may also be the case that elimination of indolent populations actually promotes survival of aggressive populations. In either event, clinical “transformation” typically occurs in the absence of therapy.

**Fig 3:**
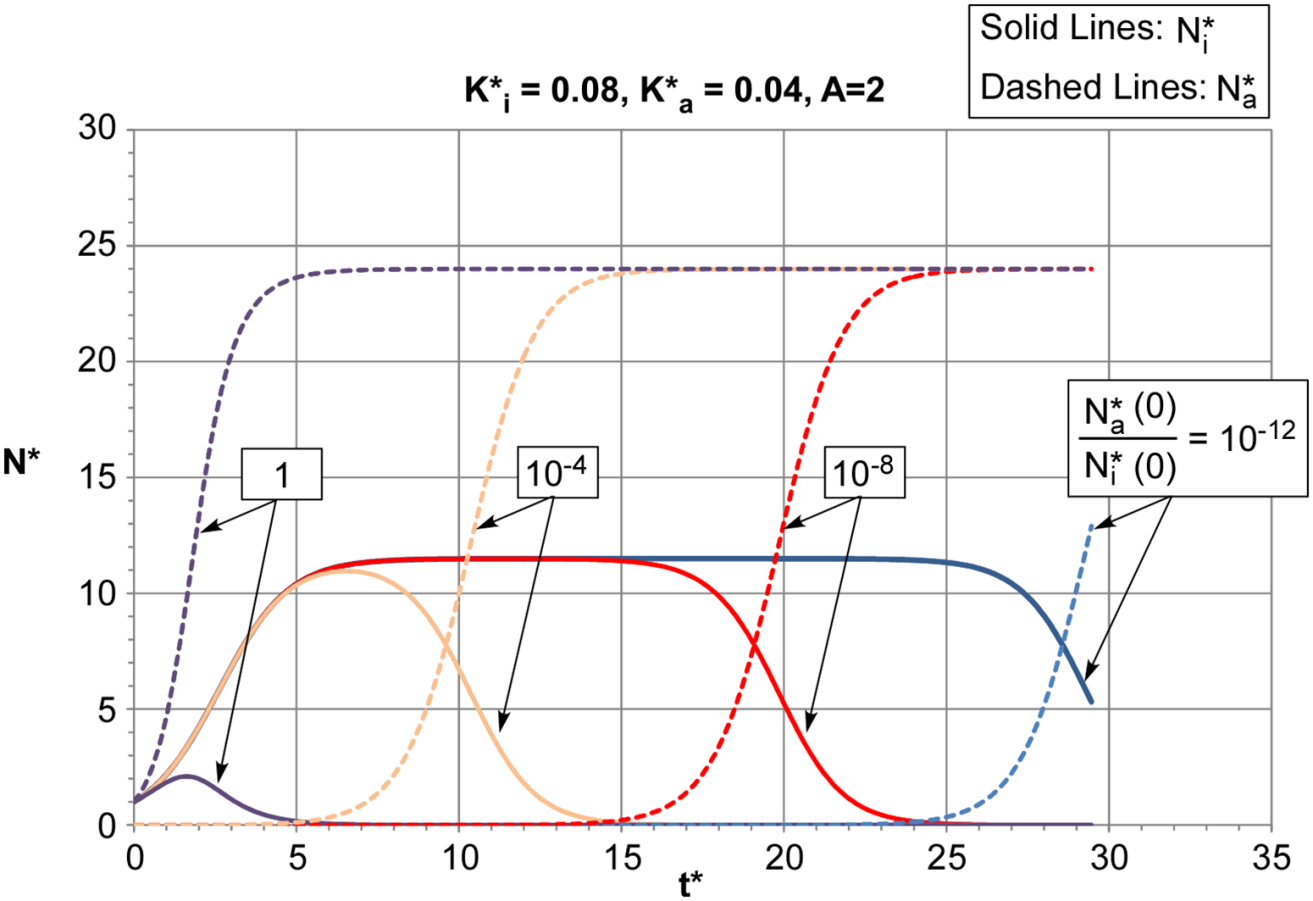
Populations of aggressive B-cells, N*_a_, and indolent B-cells, N*_i_, vs time and initial ratios N*_a_(0)/N*_i_(0).

We model disease transformation ***due to an initially heterogeneous population of malignant cells*, *without considering the possibility of genetic mutations that can transform indolent cells to aggressive ones***. This may be important for initial treatment protocols. Assuming an initial ratio of the aggressive cell population to indolent cell population, the population histories of the two types can be computed. Examples of these results as a function of non-dimensional time are shown in Fig 3. In each case, we designate disease “transformation” as the time when the aggressive population overtakes the indolent population. In these examples, the aggressive cell birth rate is taken to be twice that of the indolent cells.

**Fig 4:**
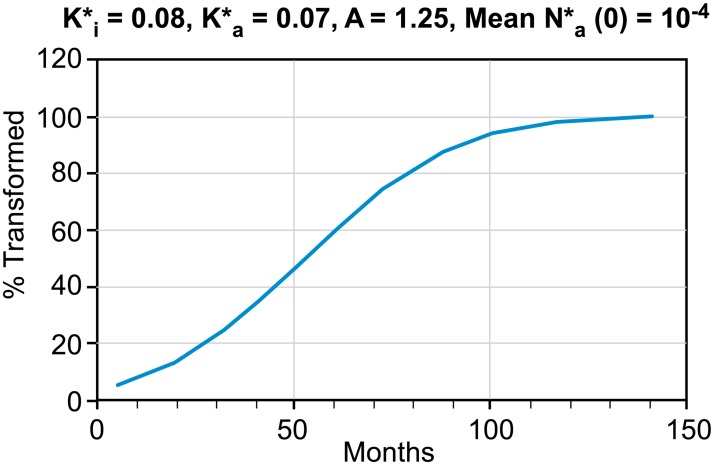
Cumulative per cent of heterogeneous populations transformed vs. time.

To develop a prediction of the probability of transformation we assume a statistical distribution of the initial aggressive cell population. Because of the large range that the ratio of initial aggressive to indolent cell populations can present (from 100% aggressive to a single cell; i.e., one cell in 10^12^), we will assume a log normal distribution. We than select values from this distribution and compute the time at which the two cell populations intersect (e. g. the transformation time). These results can then be used to calculate the cumulative percentage of indolent disease transformed to aggressive disease, as shown in Fig 4.

For this calculation we have assumed a mean value of the initial aggressive population to total population ratio of 10^–4^ and a variance of 1.0. In addition to the parameter values indicated in Fig 4, we have assumed a characteristic cell lifetime for humans of two months [8]. The results are most sensitive to A (the ratio of cell birth rates) and the mean value of the initial cell distribution ratio. The cumulative percentage of the initially heterogeneous cell population transformed asymptotes to 100% of those with *at least* a single aggressive cell at the initial time of an experiment or diagnosis, and *not* of the entire population.

Fig 4 illustrates that even a single aggressive cell in a mixed cell population can dominate a heterogeneous population in 120 months, or 10 years. Variation of parameters reveals that transformation can occur, and be observable, as late as 15 years, which is consistent with clinical observations. Furthermore, if 30% of an initial cohort of indolent NHL patients ultimately transforms to aggressive NHL, the average rate of transformation of 2% per year is also consistent with clinical experience. If our hypothesis is correct, the insignificant incidence of transformed NHL beyond 15 years is not surprising.

## Approximate Model

An approximate analytic solution to Eq (6a) and (6b) provides further insight into the influence of critical parameters such as A, the ratio of aggressive to indolent cell birth rates. With no drug therapy [f (d*) = 0], Eq (6a) and (6b) become:

$${\text{dN*}}_{\text{i}}{\text{/dt* = N*}}_{\text{i}}{\text{ \{ 1- K*}}_{\text{i}}{\text{ [1+N*}}_{\text{i}}{\text{ +N*}}_{\text{a}}\text{] \}}$$ (7a)

$${\text{dN*}}_{\text{a}}{\text{/dt* = AN*}}_{\text{a}}{\text{ \{ 1- K*}}_{\text{a}}{\text{ [1 + N*i + N*}}_{\text{a}}\text{ ] \}}$$ (7b)

At early time we expect both N*_i_ and N*_a_ to be small compared to unity. Consequently it is reasonable to linearize these equations by dropping their quadratic terms. This approximation yields:

$${\text{ln[N*}}_{\text{i}}{\text{(t*)/N*}}_{\text{i}}{\text{(0)] \textasciitilde{} (1-K*}}_{\text{i}}\text{)t*}$$ (8a)

$${\text{ln[N*}}_{\text{a}}{\text{(t*)/N*}}_{\text{a}}{\text{(0)] \textasciitilde{} A(1-K*}}_{\text{a}}\text{)t*}$$ (8b)

We expect that K*_a_ < K*_i_ because aggressive B-cells should have larger birth rates, and cell death rates should be approximately the same for all malignant B-cells. Both must be less than unity if the populations are to increase. Finally, A must be greater than unity, as aggressive cell birth rates are greater than indolent cell birth rates.

Numerical simulations of Eq (7a) and (7b) suggest that Eq (8a) and (8b) may be reasonable approximations within an interesting region of parameter space even when N*_a_ = N*_i_. Assuming this to be the case, an approximate solution for the transformation time, T*, e. g. the time it takes for the initially smaller aggressive cell population to match (and overtake) the indolent cell population, may be derived by subtracting (8a) from (8b). We find that:

T* ~ ln [N*i (0) / N*a (0)] / [A (1-K*a)-(1-K*)i] (9)

As previously noted, we expect all malignant cell death rates to be approximately equal.

If we make this assumption (K_ad_ ~ K_id_), then:

$${\text{K*}}_{\text{a}}{\text{ = K}}_{\text{ad}}{\text{/K}}_{\text{ab}}{\text{ = (K}}_{\text{ib}}{\text{/K}}_{\text{ab}}{\text{) (K}}_{\text{ad}}{\text{/K}}_{\text{ib}}{\text{) \textasciitilde{} (K}}_{\text{ib}}{\text{/K}}_{\text{ab}}{\text{) (K}}_{\text{id}}{\text{/K}}_{\text{ib}}{\text{) = K*}}_{\text{i}}\text{/A}$$ (10)

and Eq (9) reduces to

$${\text{T*\textasciitilde{} ln[N*}}_{\text{i}}{\text{(0)/N*}}_{\text{a}}\text{(0)]/(A-1)}$$ (11)

We see that the disease never transforms within a reasonable time if A ~1, as expected, because growth rates are approximately identical. When A>>1 the transformation time scales as 1/A, which is also consistent with higher birth rates of aggressive B-cells. Perhaps the most important result is that T* scales as the natural log of the ratio of initial indolent to aggressive populations.

Table 1 provides a comparison of the approximate model with an accurate numerical determination of the transformation time, T*, for various values of A and the initial conditions. The comparison between the approximate and exact values is excellent, suggesting that Eq (11) is a reasonable approximation for at least the earlier times of disease transformation.

**Table 1:** Comparison of Approximate and Exact Values of Transformation Time T*.

N*_a_(0) / N*_i_(0)	A	T*(approximate)	T*(exact)
10^−12^	2.0	28	29
10^−12^	1.5	56	52
10^−8^	2.0	18	19
10^−8^	1.5	36	34
10^−12^	3.0	14	15
10^−8^	3.0	9	10

## Immune Response

The prior sections assumed that an immune response was absent. The influence of T-cells and immunotherapies are modeled in this section. For these calculations, we use the three cell model of Eqs (3), (4) and (5) which describe a scenario that includes aggressive (N*_a_) and indolent (N*_i_) populations as well as an active immune system (N*_T_).

**Fig 5:**
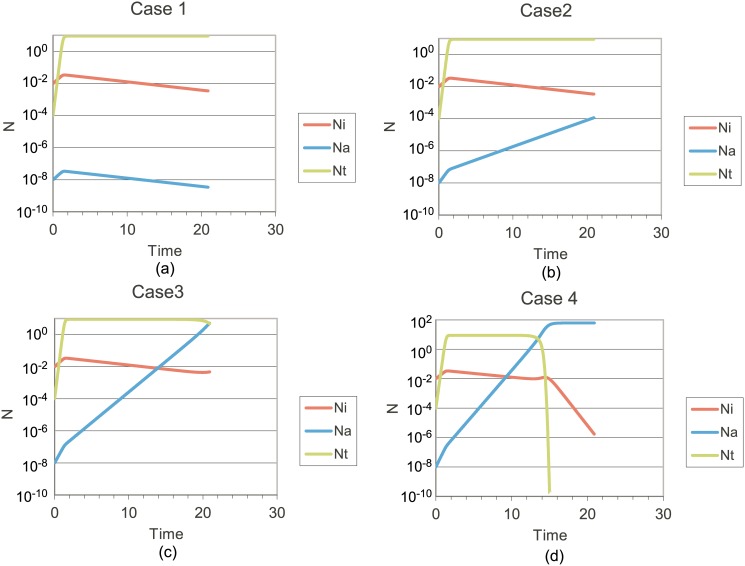
(a) Cell population histories with A = 1, where T-cells dominate both indolent and aggressive B-cell populations; (b) A = 1.5, where T-cells dominate indolent but not aggressive malignant B-cells; (c) A = 2, illustrating aggressive B-cells overtaking indolent B-cells; (d) A = 2.5, showing that aggressive B-cells have eliminated the T-cell population.

Four cases are presented in Fig 5, each illustrating a possible set of cell population histories. In all cases, we have set K*_i_ = 0.04, K*_a_ = K*_i_/A, and K*_T_ = 1/B; K'' = 0, K''' = 2, f (d*) = 1, and B = 10. These values are consistent with prior predictions of the response to immunotherapy for indolent lymphomas [5]. We then vary A, the ratio of aggressive to indolent malignant B-cell birth rates. Initial populations of all cells are indicated.

There is obviously a three-way competition between indolent and aggressive B-cells and between B-cells and T-cells. The dominant population will be determined by the relative magnitudes of parameters A and B, as wells as K'''. When therapeutic measures are introduced, parameters K', K'' and f (d*) are introduced, leading to a large array of possible outcomes.

Transformation from indolent to aggressive disease can occur at any time in sufficiently aggressive heterogeneous populations, but the scenario illustrated by Fig 5(d) may be most representative of clinical observations. This tentative conclusion will be a function of the parameters that have been fixed at specific values, but the qualitative behavior of our three-cell model is not expected to change unless immunotherapies are more effective than assumed; i.e., K'''>>2.

## Discussion

We have shown that our parametric model can be applied to aggressive as well as indolent clones of B-cell lymphomas, and that it can predict the efficacy of a combination of anti-Bcl-2 ABT-263, a predecessor drug to ABT-199, and anti-CD-20 rituximab in the absence of an immune system.

Recognizing that malignant B-cell lymphomas are inherently heterogeneous, with combinations of indolent, aggressive and very aggressive cell types resulting in more than sixty sub-classes of these diseases, we have introduced a statistical model of disease transformation that predicts both the rate of transformation and its relationship to the underlying disease parameters, and which is not inconsistent with clinical observations. An approximate analysis indicates the dependence of the time to transformation on the ratio of aggressive to indolent cell birth rates and initial populations of each cell type.

The impact of transformation on disease survival is obvious, and should be considered in initial diagnosis and treatment strategies. Our hypothesis suggests a new paradigm in which transformation is not a stochastic event that occurs due to a late “second hit”, but is rather an event predictable from sub-clone populations present at diagnosis. This would account for the counter-intuitive observation that early treatment to reduce the population of cells at risk for a second hit has not reduced the incidence of transformation. The model also accounts for the poor prognosis of transformed lymphoma in the setting of previously treated lymphoma/CLL, since the aggressive clone would then have been present during, and relatively resistant to, prior therapy. Our model also predicts that risk of transformation would not be enhanced by treatment-induced mutations, consistent with accumulating data, at least in CLL [9, 10]. The model does not rely upon, but also does not rule out, a theoretical scenario in which general cytoreduction permits faster repopulation with more aggressive subclones [1]. A corollary additionally favoring the more aggressive clones would be if that clone is also more resistant to therapy, and therefore selected, by prior therapy. In some ways, this is reminiscent of the Norton-Simon model [11] that cells in smaller tumors are more difficult to kill, although the Norton-Simon hypothesis based this on Gompertzian growth curves rather than sub-clonal differences in resistance or growth kinetics.

Identifying the subclonal driver mutations that account for transformation would allow stratification of patients at diagnosis for risk of transformation and quantitative prediction of the timing of this clinical event. Ultimately, pro-active therapeutic targeting of this clone and molecular monitoring of benefit is envisioned. These are experimentally testable in the current era of deep sequencing, where transformed lymphomas can be traced back to sub-clones present at diagnosis, and ultimately would allow us to determine if new therapies might eradicate clones with high-risk genetic features before they can become clinically evident.
